# Coffee By-Products: An Overview of Their Antimicrobial Properties

**DOI:** 10.3390/molecules31101768

**Published:** 2026-05-21

**Authors:** Sara Maia, Helena Ferreira, Maria Beatriz P. P. Oliveira, Rita C. Alves

**Affiliations:** 1 LAQV-REQUIMTE, Associated Laboratory for Green Chemistry of the Network of Chemistry and Technology, Faculty of Pharmacy, University of Porto, Rua Jorge Viterbo Ferreira 228, 4050-313 Porto, Portugal; 2 Associate Laboratory Institute for Health and Bioeconomy—i4HB, Research Unit on Applied Molecular Biosciences-UCIBIO, Faculty of Pharmacy, University of Porto, Rua Jorge Viterbo Ferreira 228, 4050-313 Porto, Portugal

**Keywords:** agro-industrial by-products, sustainability, bioactive compounds, health applications, valorization, circular economy

## Abstract

Coffee is among the most widely consumed beverages globally being cultivated in nearly 80 countries. Its processing generates large quantities of by-products, including mucilage, pulp/husks, silverskin, parchment, and spent coffee grounds. Although traditionally treated as waste, these residues are increasingly recognized as valuable resources rich in bioactive compounds exhibiting antioxidant, antimicrobial, and health-promoting properties. This review explores the antimicrobial potential of coffee by-products, with particular emphasis on their chemical composition and mechanisms of action. Compounds such as caffeine, chlorogenic acids, polyphenols, and melanoidins have demonstrated inhibitory effects against a broad spectrum of bacteria, including both Gram-positive and Gram-negative bacteria. Many of these compounds, which originate from plant’s defensive system or result from Maillard reactions, are known to disrupt microbial membranes, inhibit DNA repair, and interfere with pathogen metabolism. However, the available literature on their antimicrobial effectiveness remains limited. In the context of the rising worldwide concern over antimicrobial resistance, coffee by-products represent a sustainable and promising source of novel antimicrobial agents. Their valorization may support advances in food preservation, pharmaceutical innovation, and waste management practices, contributing to the implementation of a circular economy framework in the coffee industry while promoting environmental, economic, and social sustainability.

## 1. Introduction

**Figure 1:**
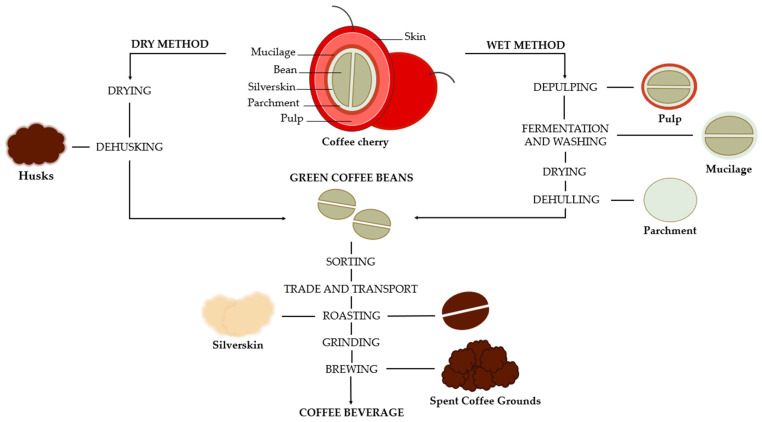
Structure of the coffee cherry and by-products derived from coffee processing.

Coffee is among the most widely consumed and produced beverages worldwide. It is cultivated in around 80 countries, representing a significant sector in the global economy, with production reaching around 173.1 million bags of 60 kg in 2023 [1]. The main coffee trade is made with two species, *Coffea arabica* (Arabica) and *Coffea canephora* (Robusta). Globally, 60% of coffee production comes from the Arabica species, primarily grown in Colombia, Brazil, and certain Asian countries like India, as well as in African nations such as Kenya and Ethiopia. The remaining 40% consists of the *Canephora* species, mainly cultivated in Africa, Brazil, and Indonesia. Arabica and Robusta differ in terms of geographical distribution, genetics, origin, physiology, and phenology, which will have a direct impact on their chemical composition and sensory characteristics. Arabica coffee plants require specific conditions to grow successfully. They flourish at higher altitudes, cooler climates, and are very susceptible to pests, diseases, and environmental stress. In contrast, Robusta coffee is much more adaptable to diverse environments. It thrives at lower altitudes and can withstand warmer climates and harsher growing conditions. Additionally, it shows increased rust resistance and higher levels of caffeine [2,3,4]. These differences in chemical composition directly influence their taste. Arabica is known for its delicate and complex flavor, often described as sweet, and complex. On the other hand, Robusta tends to have a more intense and bitter flavor. The set of these characteristics results in different industrial applications, market segments, and prices depending on the coffee species. Arabica coffee dominates the specialty coffee market, being typically sold at higher prices. On the other hand, Robusta is widely used in mass-market coffee products [5]. Coffee plants are cultivated predominantly in the region commonly referred as the “coffee belt”, located among the Tropic of Cancer and Capricorn [6]. Around 60 countries within this zone produce coffee, meaning that most coffee production comes from developing countries [2]. However, coffee consumption is much higher in developed countries [1]. This pattern may be associated with multiple aspects, including consumer consciousness about the potential health benefits of coffee [7]. The coffee cherry consists of two beans at its core, surrounded by silverskin, parchment, mucilage, pulp, and an outer skin. An overview of the coffee processing, from the coffee cherry structure to final beverage, is presented in Figure 1, highlighting the main by-products generated during processing. However, only the beans are used in the preparation of coffee beverages [8]. This transformation of coffee cherries into roasted beans requires an elevated degree of processing that involves two primary processing methods: the wet method and the dry method [9]. These processes generate significant amounts of residues, including mucilage, pulp or husks, defective beans, silverskin, parchment, and spent coffee grounds (SCGs) (Figure 1). For every ton of cherries, approximately 200 kg of green coffee beans are obtained. The dry method is the most traditional because, in addition to being simpler, it is the least polluting as it produces a smaller quantity of by-products, with coffee husk representing the main by-product (Figure 1). Coffee husks constitute approximately 12% and 18% of the dried coffee cherry [10]. In contrast, the wet method requires substantial amount of water and produces more by-products, mainly pulp (39%), mucilage (22%), and parchment (39%) (Figure 1) [10,11]. However, as the components are better preserved, it produces higher quality coffee beans, and the amount of defective beans is lower [10]. Most of these by-products end up discarded in landfills, contributing to serious environmental issues such as soil contamination [9,12], which puts the sustainability of the coffee supply chain at risk, not just environmentally, but also socially and economically. Environmentally, it is crucial to reevaluate those waste materials, repurposing them to close the loop in a zero-waste system. The overexploitation of natural resources has led to depletion rates that exceed their natural capacity for regeneration, highlighting the need for effective resource management. Such strategies are essential to prevent deforestation, protect habitats of endangered species, promote sustainable agricultural practices, and reduce pollution [13]. Economic and social sustainability focuses on achieving both social and economic benefits, to drive long-term economic growth, while also benefiting the environment [13]. Given the expected increase in coffee production in the coming years, it is crucial to find ways to utilize and apply coffee by-products industrially, as the coffee industry generates large quantities of nutrient-rich waste [9]. The valorization of these by-products will transform waste into economic value, which will ultimately create jobs and contribute to a more environmentally friendly industry.

Recent research indicates that coffee by-products are a valuable source of nutrients and may contribute to health promotion, as they are rich in carbohydrates, proteins, lipids, ashes, and several bioactive compounds (Table 1). This composition emphasizes the importance of developing efficient extraction methods to enable the recovery of compounds with potential uses across the pharmaceutical, cosmetic, and food sectors [9,14]. In this context, coffee cherry pulp has recently been recognized as a novel food, reflecting its nutritional properties, associated health benefits, and its longstanding traditional use in coffee-producing countries [15]. Some by-products have been identified as rich sources of bioactive compounds, particularly antioxidant molecules, such as caffeine, chlorogenic acid (CGA), polyphenols, and melanoidins. These compounds have been linked to multiple beneficial health effects, including a reduced risk of cancer, fatty liver disease, cardiovascular diseases and metabolic disorders. Moreover, they exhibit antimicrobial activity against both Gram-positive and Gram-negative bacteria, such as *Staphylococcus aureus*, *Bacillus subtilis*, *Pseudomonas aeruginosa*, *Enterococcus faecalis* and *Escherichia coli* [16,17,18,19].

The bioactive properties of coffee are influenced by several factors, including species, roasting degree, brewing conditions, and decaffeination processes, which may lead to variability in antimicrobial effectiveness [18]. The antimicrobial activity of coffee is mainly associated with the presence of phenolic compounds, caffeine, and Maillard reaction products, particularly melanoidins. These compounds are generated during roasting through caramelization and Maillard reactions and, depending on their concentration, can interfere with the integrity and functionality of the cell membrane, as well as inhibit DNA repair mechanism, ultimately suppressing bacterial growth [53,54]. Among phenolic compounds, several groups are known to contribute significantly to antimicrobial activity, such as phenolic acids, hydroxycinnamic acids, tannins, malic acids and flavonoids. This antimicrobial effect is believed to be a plant’s natural defense mechanisms against pathogens, herbivores, and environmental stress [55]. Moreover, caffeine, a plant alkaloid known for its chemical defense properties, has been found to exhibit antioxidant properties [56], antibacterial [57], and antimicrobial effects against several microorganisms [39,58]. Nowadays, antimicrobial resistance is becoming an increasing global concern, affecting both healthcare and agricultural sectors, as it reduces the effectiveness of widely used antibiotics in the treatment of diverse diseases. This growing resistance is further exacerbated by the overlapping use of similar antimicrobial agents across both sectors. Consequently, it is essential to explore discover novel and efficient antimicrobial compounds to support antibiotic therapy. In this regard, the valorization of natural resources is considered a promising strategy [59,60]. Despite the growing body of literature on coffee by-products, current knowledge remains fragmented, particularly regarding their antimicrobial potential. Despite the growing number of studies reporting bioactive compounds and their biological activities, a comprehensive and critical synthesis focusing specifically on antimicrobial properties, mechanisms of action, and comparative effectiveness across different coffee by-products is still lacking. In addition, important aspects such as the influence of extraction methods, concentration-dependent effects, and limitations for real-world applications remain insufficiently addressed. Therefore, this review focuses on the antimicrobial properties of coffee by-products, highlighting their chemical composition, mechanisms of action, and effectiveness against different microorganisms. Furthermore, this work seeks to compare findings across studies, identify current limitations, and discuss future perspectives for the valorization of these materials.

## 2. Materials and Methods

**Figure 2:**
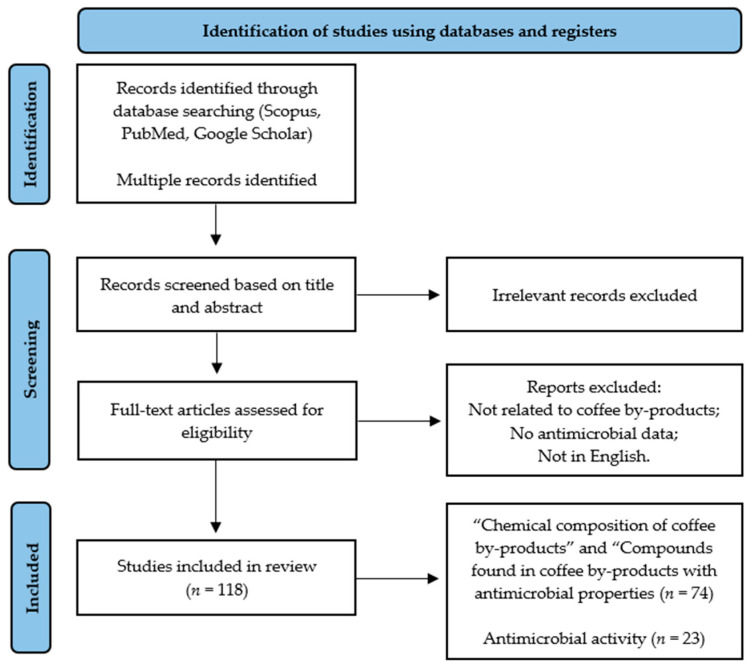
Diagram of the literature selection process.

This comprehensive review was performed through a literature search in three databases: Scopus, PubMed, and Google Scholar. The search included studies published between 2010 and 2025, although some earlier relevant studies have also been included to avoid overlooking important information. The literature search was performed using combinations of keywords related to coffee by-products and their antimicrobial properties, including: “coffee”, “by-products”, “pulp”, “parchment”, “silverskin”, “husk”, “mucilage”, “spent coffee grounds”, “defective beans”, “chemical composition”, “bioactive compounds”, “phenolic compounds”, “caffeine”, “chlorogenic acids”, “polyphenols”, “antimicrobial”, “antibacterial”, “antifungal”, “sustainability”, and “health”. Studies were included according to established inclusion and exclusion criteria. Inclusion criteria comprised studies addressing the chemical composition of coffee by-products, the presence of bioactive compounds, and antimicrobial activity, including in vitro or in vivo evaluations against microorganisms. Studies focusing on the valorization of coffee by-products for food, pharmaceutical, or agricultural applications were also considered. Exclusion criteria included studies lacking experimental evidence of antimicrobial activity, studies focused exclusively on coffee beverages or beans without reference to by-products, and publications not available in English. The selection procedure consisted of an initial screening of titles and abstracts, followed by a full-text evaluation of potentially relevant studies. After removing non-relevant studies, a total of 118 articles were included in this review. Among these, 74 studies were used for the sub-chapters “Chemical composition of coffee by-products” and “Coffee by-products as a source of antimicrobial compounds”, while 23 studies specifically addressed main objective of this review, exploring the antimicrobial activity of coffee by-products. Some articles were included in both categories, as they reported on multiple aspects of coffee by-products. To improve methodology transparency, a diagram summarizing the study selection process is presented in Figure 2.

## 3. Chemical Composition of Coffee By-Products

The composition of coffee by-products varies according to the coffee species and several external conditions. Their composition is strongly affected by the processing method, roasting level, and brewing technique [18,61]. The chemical profile of the different by-products generated along the coffee production chain is presented in Table 1. For certain by-products, such as mucilage, parchment, and defective coffee beans, their chemical composition remains largely unknown or insufficiently studied. However, as shown in Table 1, they typically contain high levels of dietary fiber, carbohydrates, caffeine and phenolics [62]. In comparison, *Coffea arabica* shows higher levels of lipids and sucrose than *Coffea canephora*. In contrast, Robusta is characterized by its greater levels of ashes, polysaccharides, chlorogenic acids and caffeine [63]. The type of processing method influences the nature of the by-products generated. The dry method, commonly applied to Robusta coffee, predominantly results in the formation of husks, while the wet method, typically associated with Arabica coffee, mainly produces pulp as a by-product (Figure 1). One of the primary by-products obtained from coffee cherry processing is coffee pulp (CP), representing about 39% of the fruit mass. It is rich in soluble fibers, proteins, carbohydrates, minerals, and numerous bioactive compounds, including caffeine, protocatechuic acid, ferulic acid, 5-caffeoylquinic acid (5-CQA), 3-p-coumaroylquinic acid, 3-feruloylquinic acid (3-FQA), flavan-3-ols, hydroxycinnamic acids, epicatechin, and catechin, which contribute to its antimicrobial and antioxidant properties [19,59,64,65,66,67]. Chlorogenic acids are the predominant compounds in CP, making up approximately 42% of its total phenolic acids [9,59,66,68,69]. Studies have shown that the aqueous extract of coffee pulp is effective in inhibiting Gram-positive bacteria like *S. aureus* [59,70]. Coffee mucilage is a translucent layer made of pectic substances, cellulose, and non-cellulosic polysaccharides, representing around 14% of the fruit’s dry weight [71,72]. The composition of coffee mucilage includes mainly water, proteins, and carbohydrates. CGA and caffeine are the most prominent bioactive compounds, but phenolics such as flavonoids and tannins are also present in the mucilage [43,59,73]. Ethanolic extracts of mucilage have demonstrated effectiveness against Gram-positive bacteria like *Bacillus cereus* [59,68].

Coffee parchment corresponds to the lignocellulosic endocarp that surrounds the seed and serves as a natural barrier between them. It represents approximately about 39% of the fruit and is rich in dietary fiber while having a relatively low-fat content [40,41,74]. The dietary fiber profile is composed entirely of the insoluble fraction, mainly composed by hemicelluloses, α-cellulose, and pectic polysaccharides [39]. The limited available information indicates that parchment contains high levels of caffeine and phenolic compounds [75,76]. CGA represent the major phenolic fraction in coffee parchment; however, other hydroxycinnamic acids, like *p*-coumaric (*p*-CoQA) and caffeic acid, also contribute to its phenolic profile [75]. These compounds have been associated with the antimicrobial properties observed in coffee-derived materials [39]. In contrast, coffee husk, produced by the dry method, consists of a combination of pulp, mucilage, and parchment, and outer skin. It is the main by-product of this process, representing approximately 45% of the fruit’s dry weight [59,77]. From a compositional perspective, coffee husk has high levels of dietary fiber, carbohydrates, and protein. It also contains a considerable amount of organic compounds, like chlorogenic acids, phenolic acids, malic acid, hydroxycinnamic acid, tannins, and caffeine, contributing to their antimicrobial and antioxidant properties [59,78,79,80]. Coffee silverskin consists of a thin, papery layer enclosing the coffee seed within the coffee cherry [10]. It is rich in dietary fiber, polysaccharides, proteins, and ash, along with a low-fat content [28,29,30,81]. During the roasting process, high temperatures promote the transformation of sugars into hemicellulose and cellulose, which constitute its main components [31,62]. In silverskin, CGA are the predominant phenolics, particularly 5-CQA [82,83]. However, other CGAs, such as 3-FQA, 5-feruloylquinic acid (5-FQA), 3-caffeoylquinic acid (3-CQA), 4-caffeoylquinic acid (4-CQA), and dicaffeoylquinic acids (di-CQA), have also been reported [84]. Antioxidants such as caffeine, trigonelline and melanoidins are also part of silverskin’s bioactive compounds. Melanoidins are also products of the high temperatures involved in the roasting process [81,85,86]. The chemical composition of defective green coffee beans (black, sour, and immature beans), differs from non-defective beans [44]. Caffeine content in green coffee varies according to species, with robusta typically containing approximately twice the amount found in Arabica. However, no significant differences in caffeine content have been reported among the different types of defective coffee beans [44]. However, in *Coffea arabica* defective beans, caffeine levels are about 0.9% higher compared to non-defective beans [44,87,88]. The CGA levels in green coffee beans vary by type, with immature beans containing 4.43%, black and sour beans both at 4.26%, and non-defective beans at 4.07% [44]. Defective green coffee beans contain substantial amounts of dietary fiber, representing around 56% of their dry weight [32]. Spent coffee grounds (SCG) are the final by-product generated, accounting for nearly 90% of the initial coffee bean mass [10,89,90]. These residues are formed during brewing, as roasted coffee is exposed to hot water or steam extract soluble compounds for beverage preparation. As a result, SCG are enriched in melanoidins and sugars such as galactose, glucose, arabinose, and mannose, which are incorporated into cellulose and hemicellulose structures [47,48,91]. The high temperatures of roasting also lead to the conversion of a portion of CGA into quinolactones [69]. Besides polysaccharides, SCG also contain proteins, minerals, dietary fiber, and lipids, including substantial amounts of palmitic, linoleic acids, and vitamin E [31,48,92]. SCG extracts have been reported to present a rich profile of phenolic compounds, including tannic, chlorogenic, gallic, caffeic, ellagic, ferulic, *p*-coumaric, and protocatechuic acids. They also contain flavonoids such as quercetin, catechin, epicatechin, and rutin, along with alkaloids, like trigonelline and caffeine. Furthermore, diterpenes including cafestol and kahweol have also been identified in SCG extracts [92,93,94,95,96].

### 3.1. Coffee By-Products as a Source of Antimicrobial Compounds

Although coffee is mainly valued for its taste and neurostimulation effects, it also possesses health-promoting properties. Among the diverse range of molecules present in coffee by-products, phenolics, alkaloids and melanoidins are particularly relevant. Together, these compounds contribute to the antimicrobial properties associated with coffee [59]. These effects are linked to bioactive molecules produced through the plant’s secondary metabolism, which primarily function as natural defense mechanisms against pathogens and environmental stressors [59].

#### 3.1.1. Phenolic Compounds

Phenolic compounds are defined as molecules containing one or more hydroxyl groups bonded to an aromatic ring. They can be broadly divided into simple phenolics, containing a single phenolic unit, and polyphenols, which are composed of multiple phenolic structures [59]. In coffee by-products, polyphenols are the predominant group, particularly hydroxybenzoic and hydroxycinnamic acids. Representative hydroxybenzoic acids found in coffee include gallic and syringic acids [92]. In addition, hydroxycinnamic acids such as caffeic, ferulic, *p*-coumaric, and chlorogenic acids are also commonly identified (Table 2) [92,97]. Among these, chlorogenic acids, formed through the esterification of quinic and caffeic acids, are especially relevant (Table 2) [68]. Over 40 derivatives of chlorogenic acid have been reported in coffee beans, including CQA, di-CQA, and FQA [63,98,99]. The antimicrobial properties of coffee by-products are strongly related to these compounds, which are considered one of the main bioactive components [100]. Flavonoids such as catechin, epicatechin, and quercetin are also present (Table 2) and contribute to the overall antimicrobial potential [101,102,103]. Catechin, commonly found in green tea, has been reported to inhibit the growth of pathogenic microorganisms like *Streptococcus mutans* [104]. Comparably, quercetin, has shown antimicrobial activity against both Gram-positive and Gram-negative bacteria, including the suppression of biofilm formation [59,105].

#### 3.1.2. Alkaloids

Alkaloids constitute a class of bioactive compounds present in coffee. Among them, caffeine stands out as the most prominent (Table 2). Caffeine belongs to the purine group of alkaloids and contributes to coffee’s bitter taste [97,106]. Another important alkaloid is trigonelline, a pyridine-derived compound formed through the enzymatic methylation of nicotinic acid, which contributes to the characteristic bitterness of coffee. Both caffeine and trigonelline concentration are affected by factors like the coffee species and growing conditions. Some studies suggest that these alkaloids contribute to the antimicrobial properties of coffee [106]. In particular, caffeine has shown potential to boost the effectiveness of certain antibiotics, enhancing their ability to kill specific bacteria when used together [68,107,108].

#### 3.1.3. Melanoidins

During roasting, green coffee beans undergo major chemical transformations. The high temperatures and low moisture conditions associated with this process promote the Maillard reaction [109]. In its final stages, this reaction leads to the formation of melanoidins, which are complex polymeric compounds produced through interactions between amino groups (from vitamins, amino acids, or proteins) and carbonyl groups derived from reducing sugars or oxidized lipids [84]. These compounds are responsible for the dark coloration, as well as the characteristic texture and flavor of foods exposed to high temperatures, such as those observed during coffee roasting. Research indicates that they contribute to coffee’s antioxidant properties and metal chelating capacity, which may explain its documented antibacterial and antioxidant [109,110]. The coffee by-product with the highest content of coffee melanoidins (CM) is typically SCG [20] (Table 2). Coffee silverskin also contains some melanoidins since it comes off during roasting, but in lower quantities than SCG (Table 2). SCG combined with CM have demonstrated notable antimicrobial effects against *S. aureus* and *E. coli*. Interestingly, when tested individually, CM demonstrated antimicrobial activity 2 to 5 times greater than when combined with SCG, suggesting that certain components of SCG may reduce the effectiveness of CM [20,29]. This reduction may be related to the chelating ability of CM, which is considered a key mechanism behind their antimicrobial properties [111]. In other words, interactions between CM and the SCG matrix could limit this chelating activity, ultimately reducing their antimicrobial potential [29].

## 4. Antimicrobial Activity of Coffee By-Products

Antimicrobial agents are substances that can destroy microorganisms or inhibit their proliferation, including bacteria and fungi [77]. Across the last decade, a growing body of literature has consistently demonstrated that coffee by-products exhibit antimicrobial activity. This effect is largely attributed to its bioactive constituents, particularly phenolic compounds, alkaloids, and melanoidins, with effectiveness largely dependent on extract composition, concentration, processing conditions, and even bacterial cell structure. However, as presented in Table 3, it is important to note that antimicrobial activity has been evaluated using different methodological approaches, which may influence the reported outcomes and limit direct comparison between studies.

A comprehensive study by Jiménez-Zamora et al. [29], evaluated the antimicrobial potential of CS (Coffee silverskin) and SCG, with particular emphasis on their CM content. Their work demonstrated that CS and SCG without melanoidins exhibited negligible activity against *E. coli* and *S. aureus*. In contrast, SCG containing melanoidins showed significant antimicrobial activity against both bacteria, confirming CM as a key bioactive fraction. When tested alone, melanoidins were 2–5 times more active compared to when incorporated in the SCG matrix, suggesting that interactions within the matrix may interfere with the efficacy of melanoidins, potentially by reducing their chelating activity, one of the key mechanisms underlying their antimicrobial function. This study also introduced the concept of concentration dependence and Gram selectivity, as physiological concentrations of CM were effective mainly against Gram-positive bacteria, likely due to the absence of the outer membrane barrier characteristic of Gram-negative strains. Furthermore, roasting conditions were shown to modulate activity, with torrefacto roasting enhancing the antimicrobial properties of melanoidins, especially against *S. aureus*. This effect was more pronounced as the proportion of torrefacto coffee increased, suggesting that the intensity of sugar-based roasting plays a key role in boosting the antimicrobial properties of coffee melanoidins [29]. In the same year, Monente et al. [112] furthered this investigation by evaluating coffee brews and SCG extracts from Arabica and Robusta species against foodborne pathogens (*S. aureus*, *L. monocytogenes*, *Salmonella*, *E. coli*) and microorganisms responsible for spoilage (*P. aeruginosa*, *B. subtilis*, *A. niger*, *C. albicans*). The results showed a greater susceptibility of Gram-positive bacteria, particularly *S. aureus* and *L. monocytogenes*, compared to Gram-negative bacteria. SCG aqueous extracts generally exhibited stronger antimicrobial activity than coffee brews, requiring relatively low concentrations of SCG aqueous extracts to effectively inhibit Gram-positive bacteria. This trend was further supported by minimum inhibitory concentration (MIC) results, which showed that Gram-positive bacteria required lower extract concentrations for growth inhibition, presenting the lowest MIC value (5 mg FDE/mL, freeze dried extract), for *S. aureus*. This observation reinforces the importance of by-product valorization, as SCG extracts may exhibit equal or even superior antimicrobial potential compared to coffee brews. Among the Gram-positive bacteria, *B. subtilis* was the least sensitive, which is consistent with previous reports [113]. This increased resistance may be attributed to its ability to form endospores, a survival mechanism known to increase resistance to environmental stressors [114]. In contrast, higher concentrations were necessary to inhibit Gram-negative bacteria and *C. albicans*. Indeed, concentration levels ranging from 5 to 80 mg FDE/mL were required to inhibit a broad spectrum of microorganisms, with Gram-negative bacteria such as *E. coli* showing higher resistance and requiring larger extract amounts. These findings were linked to the presence of caffeine, phenolic compounds, and melanoidins, emphasizing the potential of spent coffee as an affordable natural antimicrobial source [112]. These observations are in agreement with those reported by Jiménez-Zamora et al., reinforcing the role of melanoidins and the higher susceptibility of Gram-positive bacteria across different coffee by-products.

**Table 3:** Studies showing antimicrobial activity in coffee by-products.

Reference	By-Product	Extract	Microorganism	Concentration (mg/mL)	Method	Inhibition Zone/MIC	Main Findings
Jiménez-Zamora et al. [29]	CS and SCG	Aqueous/isolated melanoidins	*E. coli*, *S. aureus*	1–10	Microtiter plate	NR	Strong antimicrobial activity associated with melanoidins; Higher effect against Gram-positive bacteria.
Monente et al. [112]	SGC	Aqueous	*S. aureus*, *L. monocytogenes*, *B. subtilis*, *E. coli*, *Salmonella choleraesuis*, *P. aeruginosa*, *A. niger*, *C. albicans*	5–160 *	Agar-well diffusion	Moderate (higher for Gram+) MIC: 5 mg/mL *	Demonstrated activity mainly against Gram-positive bacteria and yeast (*C. albicans*); No effect against *A. niger.*
Duangjai et al. [19]	Pulp	Aqueous	*S. aureus*, *S. epidermidis*, *P. aeruginosa*, *E. coli*	4.69–75 *	Agar-well diffusion	Reported (higher for Gram+) MIC: 4.69 mg/mL *	Inhibitory action against all tested bacteria; Gram-positive bacteria demonstrated more susceptibility; *S. epidermidis* was the most sensitive.
Khochapong et al. [70]	Pulp	Aqueous	*E. coli*, *S. aureus*, *L. acidophilus*	150, 200, 250 and 300 *	Disk diffusion	Qualitative (opaque zone)	Inhibition observed at 150 mg FDE/mL (*E. coli*) and 200 mg FDE/mL (*S. aureus*); no effect on probiotics; Reduced activity after digestion.
Chaves-Ulate et al. [68]	Mucilage	Ethanolic	*Alcaligenes* spp., *Serratia* spp., *M. luteus*, *E. coli*, *S. aureus*, *B. cereus*, *Salmonella enterica*, *L. monocytogenes*, *P. aeruginosa*, *L. acidophilus*, *L. casei*, *L. rhamnosus*, *Lactiplantibacillus plantarum*	6.7–50.4 *	Agar microdilution	NR	Activity against Gram-positives, especially *B. cereus*; Gram-negatives were resistant; The effect of the extract is concentration dependent.
Prasetya et al. [115]	Husks	Aqueous	*Enterococcus faecalis* and *P. gingivalis*	250, 500, 750 and 1000 *	Disk diffusion	Medium–High	Activity against both *E. faecalis* and *P. gingivalis*; The effect of the extract is concentration dependent.

NR = Not Reported; CS = Coffee Silverskin; SGC = Spent Coffee Grounds; * Values reported in mg FDE/mL, Freeze Dried Extract.

Subsequent studies shifted focus to coffee pulp, highlighting the influence of extraction and processing methods. According to Duangjai et al. [19], aqueous extracts of coffee pulp containing high levels of chlorogenic acids and caffeine exhibited strong inhibitory activity, especially on Gram-positive bacteria (*S. epidermidis* and *S. aureus*), compared to Gram-negative strains. The extract contained chlorogenic acids, malic acid, quinic acid, and caffeine as the predominant compounds, which may explain its antimicrobial activity. An MIC of 4.69 mg FDE/mL was reported against *S. epidermidis*. Furthermore, concentrations ranging from 4.69 to 75 mg FDE/mL were effective in inhibiting both Gram-positive and Gram-negative bacteria. However, minimal bactericidal concentration (MBC) results indicated that even at 300 mg FDE/mL, the extract did not exhibit bactericidal activity, suggesting a predominantly bacteriostatic action. The observed inhibition of *S. aureus* and *E. coli*, both associated with foodborne illnesses, further highlights the potential of coffee pulp as a promising candidate for application as food preservative or additive. More recently, Khochapong et al. [70] introduced a gastrointestinal perspective by evaluating aqueous extracts of coffee pulp (*Coffea arabica*) before and after simulated in vitro digestion, as phenolic compounds may degrade during digestion and potentially reduce antimicrobial efficacy. The extracts were tested against common pathogenic bacteria and a probiotic strain (*L. acidophilus*) using the disk diffusion method. Undigested extracts demonstrated inhibitory activity against both *S. aureus* and *E. coli*, with inhibition zones at 150 and 200 mg FDE/mL, respectively, but without achieving complete inhibition. In comparison, the positive controls (penicillin for *S. aureus* and ampicillin for *E. coli* and *L. acidophilus*) exhibited substantially larger inhibition zones, indicating a significantly higher antimicrobial effectiveness than the coffee pulp extracts. Moreover, antimicrobial activity decreased after digestion, in parallel with reductions in phenolic content and antioxidant capacity. This finding highlights a critical limitation, as in vitro antimicrobial activity may not directly translate to in vivo conditions due to compound instability during digestion. Notably, neither digested nor undigested extracts inhibited the probiotic *L. acidophilus*, indicating a selective antimicrobial effect that may be advantageous for functional food applications [70]. These observations, also summarized in Table 3, highlight the impact of extraction techniques and gastrointestinal stability on the antimicrobial performance of coffee by-products. Similarly, Chaves-Ulate et al. [68] reinforced the Gram-dependent susceptibility pattern using an ethanolic extract of coffee mucilage. The results indicated significant inhibition of growth among Gram-positive bacteria, including *M. luteus*, *B. cereus*, *S. aureus*, and *L. monocytogenes*, with *B. cereus* being the most sensitive. Conversely, Gram-negative bacteria (*Alcaligenes* spp., *Salmonella* spp., *E. coli*, and *Pseudomonas* spp.) were less affected. This difference in susceptibility was linked to structural variations between the two groups, particularly the outer membrane of Gram-negative bacteria, which is rich in lipopolysaccharides and acts as a barrier to antimicrobial compounds. They observed that certain phenolics can disrupt bacterial membranes and acidify the cytoplasm, ultimately causing cell death, an effect to which Gram-negative bacteria are generally more resistant. Three extract concentrations (6.7, 26.2, and 50.4 mg FDE/mL) were evaluated. The lowest concentration showed limited antimicrobial activity, inhibiting only *B. cereus*, *M. luteus*, and *L. monocytogenes*. Overall, a clear concentration-dependent effect was observed, with the highest concentration (50.4 mg FDE/mL) consistently exhibiting the strongest inhibitory activity across the tested microorganisms, suggesting that higher extract levels, not tested in this research, might overcome resistance in Gram-negative strains [59,68]. In agreement with Table 3, this study provides strong evidence of selective antimicrobial activity and highlights the importance of considering both microbial type and extract concentration when evaluating efficacy.

The most recent work by Prasetya et al. [115] extended the application of coffee by-products to oral health, demonstrating concentration-dependent antibacterial activity of coffee husk aqueous extracts against *Enterococcus faecalis* and *Porphyromonas gingivalis*. The antimicrobial activity of husk extracts was assessed by the disk diffusion method at concentrations ranging from 250 to 1000 mg FDE/mL. The data demonstrated a concentration-dependent antibacterial effect. For *E. faecalis*, all tested extract concentrations showed moderate antibacterial activity (inhibition zones between 6 and 10 mm), which remained consistently lower than the positive control. In contrast, for *P. gingivalis*, the antimicrobial effectiveness of the extract was comparable or greater than that the positive control at concentrations of 500 mg FDE/mL and above, with the highest inhibition zone (20.07 mm) observed at 1000 mg FDE/mL. However, even at lower concentrations, all tested extracts exhibited inhibitory effects, supporting the versatility of coffee husk bioactives against clinically relevant bacteria [115]. These findings may suggest potential applications beyond food systems, particularly in oral health and biomedical fields. Taken together, these studies reveal several consistent trends. First, coffee by-products tend to exhibit higher activity against Gram-positive bacteria. Second, antimicrobial activity is strongly concentration-dependent and influenced by processing conditions, including roasting intensity, extraction solvent, and even digestion. Third, phenolic compounds, alkaloids, particularly caffeine, and melanoidins emerge as the primary contributors to antimicrobial activity, acting through multiple mechanisms. In addition, a comparative analysis across studies indicates that SCG and CP are the most extensively investigated and generally exhibit stronger antimicrobial activity at lower concentrations. Coffee mucilage has also demonstrated relevant antimicrobial potential, particularly against Gram-positive bacteria, although its effectiveness remains concentration-dependent. In contrast, coffee husk extracts typically require higher concentrations to achieve comparable effects. These differences emphasize the role of chemical composition, processing conditions, and extraction methods in determining antimicrobial performance. Nevertheless, as clearly illustrated in Table 3 and Table 4, some general trends, such as higher susceptibility of Gram-positive bacteria and concentration-dependent activity, are consistently observed across different coffee by-products, extracts, and experimental approaches.

However, the heterogeneity of methodologies used across studies remains a major limitation, highlighting the need for standardized protocols to allow more reliable comparisons of antimicrobial effectiveness. Furthermore, defective coffee beans and parchment, remain poorly explored in terms of antimicrobial activity, resulting in limited available data compared to more extensively studied materials such as coffee pulp and spent coffee grounds, further restricting comprehensive evaluation across different materials. Although the antimicrobial activity of coffee by-products has been increasingly reported, their effectiveness is generally lower than conventional antibiotics, as higher concentrations are often required to achieve comparable inhibitory effects [70,115]. Nevertheless, their natural origin, lower environmental impact, and potential for synergistic interactions make them promising candidates as complementary antimicrobial agents rather than direct substitutes [57,112]. Phenolic compounds are known to disrupt microbial cell membranes by altering permeability and interfering with intracellular processes, mainly through hydrogen bonding with essential enzymes. Such effects can cause irreversible disruption of the cytoplasmic membrane, along with coagulation of intracellular components, inhibition of enzymatic activity, and ultimately cell death [70]. Alkaloids usually exhibit antimicrobial activity by interacting with bacterial compounds in DNA, disturbing its structure and interfering with enzymes like topoisomerases that are essential for bacteria replication. They also interfere with peptidoglycan synthesis, weakening the bacterial cell wall and enhancing permeability [115]. Melanoidins exert their antimicrobial activity primarily through metal chelation. By chelating essential metal ions such as Fe^3+^ and Mg^2+^, these compounds limit the access of bacteria to key nutrients necessary for their growth and survival. The anionic behavior of melanoidins destabilizes the outer membrane by chelating the stabilizing cation Mg^2+^, particularly in Gram-negative bacteria, resulting in membrane disruption and subsequent cell death. Furthermore, melanoidins can cause direct damage to bacterial membranes [111]. Despite the greater resistance of Gram-negative bacteria, various studies indicate that coffee and its by-products are capable of inhibiting both Gram-positive and Gram-negative bacteria (Table 3), particularly at higher concentrations [16,19,57,68,112,116,117,118]. Despite these promising mechanisms, it is important to highlight that most available studies are limited to in vitro assays, and further research is needed to evaluate their efficacy, safety, and stability in real food systems and in vivo conditions. Considering that coffee by-products are natural resources, their antimicrobial activity cannot be directly compared to that of antibiotics, which are medicines, highly purified and specifically considered to target microorganisms. Extracts from coffee by-products require “high” concentrations to exhibit antimicrobial effects as they are complex mixtures of bioactive compounds, and the antimicrobial efficacy is dependent on factors such as extraction method, concentration, and processing conditions, demonstrating potential, particularly, in non-medical antimicrobial applications. Overall, the accumulated evidence supports the potential of coffee by-products as low-cost, natural sources of non-clinical antimicrobial agents, with promising applications in food preservation, cosmetic formulations, health, and the development of functional products. The antimicrobial activity of coffee by-products shows promising in terms of sustainability and added value products of the coffee chain.

## 5. Conclusions

Coffee by-products present promising potential as natural sources of antioxidant and antimicrobial compounds, particularly chlorogenic acids and caffeine. Their integration into food, pharmaceutical, and agricultural systems may offer sustainable and innovative solutions to current challenges, such as antimicrobial resistance and environmental waste. The valorization of coffee residues supports circular economy practices and enables the development of value-added products. However, it is important to note that much of the evidence currently available is based on in vitro studies, which may not fully reflect their effectiveness in real-world applications. Factors such as compound stability, bioavailability, and interactions within complex matrices can significantly influence antimicrobial performance. In addition, key challenges such as toxicity, safety, pharmacokinetics, optimal dosage, regulatory affairs, and scalability, remain insufficiently explored and represent important barriers that need to be overcome before their large-scale use at an industrial level.

Despite these limitations, coffee by-products remain a promising and sustainable source of bioactive compounds with potential applications in food preservation, health and other industrial fields. Future research should focus on in vivo studies and optimization of extraction techniques to support their safe and effective application. Unlocking the full potential of coffee by-products will depend on overcoming these limitations but could ultimately play a vital role in promoting sustainability, improving public health, and contribute to a circular economy.

## Figures and Tables

**Table 1:** Chemical characterization of coffee processing by-products *.

Chemical Compounds		Pulp	Husk	Silverskin	Parchment	Mucilage	Defective Beans	Spent Coffee Grounds
Macronutrients (%)	Protein	8.74–17.37	6.80	10.90–20.60	3.10–17.40	17.00	14.50–17.00	11.20–18.80
	Lipids	0.61	1.50	1.19–6.30	0.30–4.10	-	8.10–13.60	2.29–24.30
	Carbohydrates	15.68–63.57	35.00–85.00	5.80–7.97	-	4.10–7.80	58.60	62.20–69.40
	Total sugars	9.70	26.50	0.40	-	-	-	-
	Ash	9.55	1.40–6.20	3.47–10.50	6.30	-	4.80–6.00	0.50–2.20
	Total fiber	28.00	31.86–43.00	52.00–69.80	64.30–92.60	-	56.40	47.30–60.46
	Soluble fiber	18.00	17.00	3.40–11.20	0.50	-	-	0.80–9.68
	Insoluble fiber	10.00	26.00	44.20–66.00	92.10	-	-	41.63–56.80
Bioactive compounds (mg/g)	Total phenolics	10.20	12.20	3.60–20.30	2.30	-	-	12.00
	Caffeine	2.04–10.12	6.85–12.00	0.68–9.50	1.34	-	13.40–45.16	1.94–7.88
	Flavonoids	0.60	-	-	-	-	-	-
	Tannins	-	93.00	2.48	-	-	2.46	-
	Chlorogenic acids	1.80–3.37	2.50	2.46	0.05	-	42.60–44-31	2.12–7.66
	5-CQA	1.72	0.84	0.01–3.16	-	-	-	0.40–2.64
	4-CQA	-	-	0.08–0.10	-	-	-	0.097–0.25
	3-CQA	-	-	0.15	-	-	-	0.003–0.14
	Melanoidins	-	-	-	-	-	-	8.77–11.47
References		[9,20,21,22,23]	[9,20,24,25,26,27]	[20,23,28,29,30,31,32,33,34,35,36,37,38]	[36,39,40,41,42]	[20,43]	[23,32,44,45,46]	[20,29,31,47,48,49,50,51,52]

* Values reported in dry weight.

**Table 2:** Chemical composition and relevant bioactive compounds identified in coffee by-products.

By-Product	Chemical Composition	Key Bioactive Compounds
Pulp	Carbohydrates Proteins Soluble Fibers Minerals	CGA Caffeine Epicatechin Catechin
Mucilage	Carbohydrates Water Proteins Pectin	CGA Caffeine
Parchment	Ash Cellulose Hemicellulose Lignin	CGA Caffeine Gallic acid *p*-coumaric acid
Husk	Carbohydrates Proteins Dietary Fiber	CGA Gallic acid Tannic acid Epicatechin Caffeine
Silverskin	Polysaccharides Dietary Fiber Proteins Fats Ash	CGA 3-FQA 5-CQA 3-CQA *p*-coumaric acid Caffeine Trigonelline Melanoidins
Spent Coffee Grounds	Polysaccharides Dietary Fiber Vitamin E Lignin Proteins Minerals Fats	CGA Caffeic acid Gallic acid Ferulic acid Ellagic acid *p*-coumaric acid Tannic acid Quercetin Rutin Catechin Epicatechin Caffeine Trigonelline Melanoidins
Defective coffee beans	Carbohydrates Protein Fiber Lipids Ash	CGA *p*-coumaric acid Quercetin Rutin Ferulic acid Gallic acid Caffeine Trigonelline Melanoidins

Note: Adapted from [59].

**Table 4:** Comparative overview of antimicrobial properties of coffee by-products.

By-Product	Key Bioactive Compounds	Most Susceptible Microorganisms
SCG	Phenolic compounds, caffeine, and melanoidins	Gram + bacteria (*S. aureus*, *L. monocytogenes*)
CS	Phenolic compounds and melanoidins	Gram + bacteria
Coffee pulp	Chlorogenic acids, caffeine, quinic acid, malic acid, tannins and hydroxycinnamic acids	Gram + bacteria (*S. aureus*, *S. epidermidis*)
Mucilage	Phenolic compounds and caffeine	Gram + bacteria (*S. aureus*, *B. cereus*)
Coffee husk	Polyphenols, flavonoids, tannins, saponins and alkaloids	*E. faecalis*, *P. gingivalis*

SCG = Spent Coffee Grounds; CS = Coffee Silverskin.

## Data Availability

No new data were created or analyzed in this study. Data sharing is not applicable to this article.

## Abbreviations

The following abbreviations are used in this manuscript:

CGA	Chlorogenic acids
CQAs	Caffeoylquinic acids
3-CQA	3-caffeoylquinic acid
4-CQA	4-caffeoylquinic acid
di-CQA	Dicaffeoylquinic acids
FQA	Feruloylquinic acids
3-FQA	3-Feruloylquinic acids
5-FQA	5-Feruloylquinic acids
*p*-CoQA	*p*-coumaric acid
SCG	Spent coffee grounds
CM	Coffee melanoidins
CS	Coffee silverskin
HMF	Hydroxymethylfurfural
MIC	Minimum Inhibitory Concentration
MBC	Minimum Bactericidal Concentration
NR	Not Reported
ICO	International Coffee Organization

## References

[1] ICO (2023). ICO I Annual Review 2022/2023. International Coffee Organization. https://www.icocoffee.org/documents/cy2023-24/annual-review-2022-2023-e.pdf.

[2] Díaz-Hernández G.C., Alvarez-Fitz P., Maldonado-Astudillo Y.I., Jiménez-Hernández J., Parra-Rojas I., Flores-Alfaro E., Salazar R., Ramírez M. (2022). Antibacterial, Antiradical and Antiproliferative Potential of Green, Roasted, and Spent Coffee Extracts. Appl. Sci..

[3] Panusa A., Petrucci R., Lavecchia R., Zuorro A. (2017). UHPLC-PDA-ESI-TOF/MS metabolic profiling and antioxidant capacity of arabica and robusta coffee silverskin: Antioxidants vs phytotoxins. Food Res. Int..

[4] Campuzano-Duque L., Herrera J., Ged C., Blair M. (2021). Bases for the Establishment of Robusta Coffee (*Coffea canephora*) as a New Crop for Colombia. Agronomy.

[5] Alves R., Casal S., Oliveira B. (2009). Benefícios do café na saúde: Mito ou realidade?. Química Nova.

[6] dos Santos É.M., de Macedo L.M., Tundisi L.L., Ataide J.A., Camargo G.A., Alves R.C., Oliveira M.B.P., Mazzola P.G. (2021). Coffee by-products in topical formulations: A review. Trends Food Sci. Technol..

[7] Hoseini M., Cocco S., Casucci C., Cardelli V., Corti G. (2021). Coffee by-products derived resources. A review. Biomass Bioenergy.

[8] Costa A.S.G., Alves R.C., Vinha A.F., Costa E., Costa C.S.G., Nunes M.A., Almeida A.A., Santos-Silva A., Oliveira M.B.P. (2018). Nutritional, chemical and antioxidant/pro-oxidant profiles of silverskin, a coffee roasting by-product. Food Chem..

[9] Costa A.S.G., Alves R.C., Vinha A.F., Barreira S.V.P., Nunes M.A., Cunha L.M., Oliveira M.B.P. (2014). Optimization of antioxidants extraction from coffee silverskin, a roasting by-product, having in view a sustainable process. Ind. Crops Prod..

[10] Jiménez-Zamora A., Pastoriza S., Rufián-Henares J.A. (2015). Revalorization of coffee by-products. Prebiotic, antimicrobial and antioxidant properties. LWT.

[11] Murthy P.S., Naidu M.M. (2012). Recovery of Phenolic Antioxidants and Functional Compounds from Coffee Industry By-Products. Food Bioprocess Technol..

[12] Almeida A.A.P., Naghetini C.C., Santos V.R., Antonio A.G., Farah A., Glória M.B.A. (2012). Influence of natural coffee compounds, coffee extracts and increased levels of caffeine on the inhibition of *Streptococcus mutans*. Food Res. Int..

[13] Iriondo-DeHond A., Iriondo-DeHond M., del Castillo M.D. (2020). Applications of Compounds from Coffee Processing By-Products. Biomolecules.

[14] Arango-Agudelo E., Rendón-Muñóz Y., Cadena-Chamorro E., Santa J.F., Buitrago-Sierra R. (2023). Evaluation of Colombian Coffee Waste to Produce Antioxidant Extracts. BioResources.

[15] Cavanagh Q., Brooks M.S.-L., Rupasinghe H.P.V. (2023). Innovative technologies used to convert spent coffee grounds into new food ingredients: Opportunities, challenges, and prospects. Future Foods.

[16] Rawangkan A., Siriphap A., Yosboonruang A., Kiddee A., Pook-In G., Saokaew S., Sutheinkul O., Duangjai A. (2022). Potential Antimicrobial Properties of Coffee Beans and Coffee By-Products Against Drug-Resistant *Vibrio cholerae*. Front. Nutr..

[17] Daglia M. (2012). Polyphenols as antimicrobial agents. Curr. Opin. Biotechnol..

[18] Rurián-Henares J.A., Morales F.J. (2008). Antimicrobial Activity of Melanoidins against *Escherichia coli* Is Mediated by a Membrane-Damage Mechanism. J. Agric. Food Chem..

[19] Vegro C.L.R., de Almeida L.F., de Almeida L.F., Spers E.E. (2020). Chapter 1–Global coffee market: Socio-economic and cultural dynamics. Coffee Consumption and Industry Strategies in Brazil.

[20] Pieczonka S.A., Dzemajili A., Heinzmann S.S., Rychlik M., Schmitt-Kopplin P. (2025). The high-resolution molecular portrait of coffee: A gateway to insights into its roasting chemistry and comprehensive authenticity profiles. Food Chem..

[21] Puerta Quintero G. (1998). Calidad en taza de las variedades de *Coffea arabica* cultivadas en Colombia. Cenicafé.

[22] Franca A.S., Oliveira L.S., Pan Z., Zhang R., Zicari S. (2019). Chapter 17–Coffee. Integrated Processing Technologies for Food and Agricultural By-Products.

[23] Klingel T., Kremer J.I., Gottstein V., Rajcic de Rezende T., Schwarz S., Lachenmeier D.W. (2020). A Review of Coffee By-Products Including Leaf, Flower, Cherry, Husk, Silver Skin, and Spent Grounds as Novel Foods within the European Union. Foods.

[24] Ballesteros L.F., Teixeira J.A., Mussatto S.I. (2014). Chemical, Functional, and Structural Properties of Spent Coffee Grounds and Coffee Silverskin. Food Bioprocess Technol..

[25] Peixoto J.A.B., Andrade N., Machado S., Costa A.S.G., Oliveira M., Martel F., Alves R.C. (2022). Green/Roasted Coffee and Silverskin Extracts Inhibit Sugar Absorption by Human Intestinal Epithelial (Caco-2) Cells by Decreasing GLUT2 Gene Expression. Foods.

[26] Siridevi G., Havare D., Basavaraj K., Murthy P.S. (2019). Coffee starter microbiome and in-silico approach to improve Arabica coffee. LWT.

[27] Andrade N., Peixoto J.A.B., Oliveira M.B.P.P., Martel F., Alves R.C. (2022). Can coffee silverskin be a useful tool to fight metabolic syndrome?. Front. Nutr..

[28] Alves R.C., Costa A.S., Jerez M., Casal S., Sineiro J., Núñez M.J., Oliveira B. (2010). Antiradical activity, phenolics profile, and hydroxymethylfurfural in espresso coffee: Influence of technological factors. J. Agric. Food Chem..

[29] Mesías M., Navarro M., Martínez-Saez N., Ullate M., del Castillo M.D., Morales F.J. (2014). Antiglycative and carbonyl trapping properties of the water soluble fraction of coffee silverskin. Food Res. Int..

[30] Habtamu D., Belay A. (2020). First order derivative spectra to determine caffeine and chlorogenic acids in defective and non-defective coffee beans. Food Sci. Nutr..

[31] Franca A.S., Oliveira L.S., Mendonça J.C.F., Silva X.A. (2005). Physical and chemical attributes of defective crude and roasted coffee beans. Food Chem..

[32] Mazzafera P. (1999). Chemical composition of defective coffee beans. Food Chem..

[33] Prandi B., Ferri M., Monari S., Zurlini C., Cigognini I., Verstringe S., Schaller D., Walter M., Navarini L., Tassoni A. (2021). Extraction and Chemical Characterization of Functional Phenols and Proteins from Coffee (*Coffea arabica*) By-Products. Biomolecules.

[34] Balzano M., Loizzo M.R., Tundis R., Lucci P., Nunez O., Fiorini D., Giardinieri A., Frega N.G., Pacetti D. (2020). Spent espresso coffee grounds as a source of anti-proliferative and antioxidant compounds. Innov. Food Sci. Emerg. Technol..

[35] Bomfim A.S.C., Oliveira D.M., Voorwald H.J.C., Benini K., Dumont M.J., Rodrigue D. (2022). Valorization of Spent Coffee Grounds as Precursors for Biopolymers and Composite Production. Polymers.

[36] Cosío-Barrón A.C.G., Hernández-Arriaga A.M., Campos-Vega R. (2020). Spent coffee (*Coffea arabica* L.) grounds positively modulate indicators of colonic microbial activity. Innov. Food Sci. Emerg. Technol..

[37] Martinez-Saez N., García A.T., Pérez I.D., Rebollo-Hernanz M., Mesías M., Morales F.J., Martín-Cabrejas M.A., Dolores del Castillo M. (2017). Use of spent coffee grounds as food ingredient in bakery products. Food Chem..

[38] Mussatto S.I., Machado E.M.S., Martins S., Teixeira J.A. (2011). Production, Composition, and Application of Coffee and Its Industrial Residues. Food Bioprocess Technol..

[39] Chacón-Figueroa I.H., Medrano-Ruiz L.G., Moreno-Vásquez M.D.J., Ovando-Martínez M., Gámez-Meza N., Del-Toro-Sánchez C.L., Castro-Enríquez D.D., López-Ahumada G.A., Dórame-Miranda R.F. (2022). Use of Coffee Bean Bagasse Extracts in the Brewing of Craft Beers: Optimization and Antioxidant Capacity. Molecules.

[40] Bevilacqua E., Cruzat V., Singh I., Rose’Meyer R.B., Panchal S.K., Brown L. (2023). The Potential of Spent Coffee Grounds in Functional Food Development. Nutrients.

[41] Benitez V., Rebollo-Hernanz M., Hernanz S., Chantres S., Aguilera Y., Martin-Cabrejas M.A. (2019). Coffee parchment as a new dietary fiber ingredient: Functional and physiological characterization. Food Res. Int..

[42] Grassino A.N., Jerković I., Pedisić S., Dent M. (2024). Hydrodistillation fractions of coffee (green and roasted) and coffee by-product (silverskin and spent grounds) as a source of bioactive compounds. Sustain. Chem. Pharm..

[43] Sierra-López L.D., Hernandez-Tenorio F., Marín-Palacio L.D., Giraldo-Estrada C. (2023). Coffee mucilage clarification: A promising raw material for the food industry. Food Humanit..

[44] Avallone S., Guiraud J.-P., Guyot B., Olguin E., Brillouet J.-M. (2000). Polysaccharide Constituents of Coffee-Bean Mucilage. J. Food Sci..

[45] Okur I., Soyler B., Sezer P., Oztop M.H., Alpas H. (2021). Improving the Recovery of Phenolic Compounds from Spent Coffee Grounds (SCG) by Environmentally Friendly Extraction Techniques. Molecules.

[46] Ramón-Gonçalves M., Gómez-Mejía E., Rosales-Conrado N., León-González M.E., Madrid Y. (2019). Extraction, identification and quantification of polyphenols from spent coffee grounds by chromatographic methods and chemometric analyses. Waste Manag..

[47] Castillo M.D., Fernandez-Gomez B., Martinez-Saez N., Iriondo-DeHond A., Mesa M.D., Farah A., Farah A. (2019). Coffee By-products. Coffee: Production, Quality and Chemistry.

[48] Dias M., Melo M.M., Schwan R.F., Silva C.F. (2015). A new alternative use for coffee pulp from semi-dry process to β-glucosidase production by Bacillus subtilis. Lett. Appl. Microbiol..

[49] Ramirez-Coronel M.A., Marnet N., Kolli V.S.K., Roussos S., Guyot S., Augur C. (2004). Characterization and Estimation of Proanthocyanidins and Other Phenolics in Coffee Pulp (*Coffea arabica*) by Thiolysis−High-Performance Liquid Chromatography. J. Agric. Food Chem..

[50] da Silva M.R., Bragagnolo F.S., Carneiro R.L., Pereira I.D.O.C., Ribeiro J.A.A., Rodrigues C.M., Jelley R.E., Fedrizzi B., Funari C.S. (2022). Metabolite characterization of fifteen by-products of the coffee production chain: From farm to factory. Food Chem..

[51] Baêta B.E.L., Cordeiro P.H.D.M., Passos F., Gurgel L.V.A., de Aquino S.F., Fdz-Polanco F. (2017). Steam explosion pre-treatment improved the biomethanization of coffee husks. Bioresour. Technol..

[52] Oliveira G., Passos C.P., Ferreira P., Coimbra M.A., Gonçalves I. (2021). Coffee By-Products and Their Suitability for Developing Active Food Packaging Materials. Foods.

[53] Bekalo S.A., Reinhardt H.-W. (2010). Fibers of coffee husk and hulls for the production of particleboard. Mater. Struct..

[54] Silva M.D.O., Honfoga J.N.B., Medeiros L.L.D., Madruga M.S., Bezerra T.K.A. (2021). Obtaining Bioactive Compounds from the Coffee Husk (*Coffea arabica* L.) Using Different Extraction Methods. Molecules.

[55] Janissen B., Huynh T. (2018). Chemical composition and value-adding applications of coffee industry by-products: A review. Resour. Conserv. Recycl..

[56] Torres Valenzuela L.S., Serna Jimenez J.A., Martínez Cortínez K., Toledo Castanheira D. (2019). Coffee By-Products: Nowadays and Perspectives. Coffee–Production and Research.

[57] Brand D., Pandey A., Rodriguez-Leon J.A., Roussos S., Brand I., Soccol C.R. (2001). Packed bed column fermenter and kinetic modeling for upgrading the nutritional quality of coffee husk in solid-state fermentation. Biotechnol. Program.

[58] Collazo-Bigliardi S., Ortega-Toro R., Chiralt Boix A. (2018). Isolation and characterisation of microcrystalline cellulose and cellulose nanocrystals from coffee husk and comparative study with rice husk. Carbohydr. Polym..

[59] Ateş G., Elmacı Y. (2019). Physical, chemical and sensory characteristics of fiber-enriched cakes prepared with coffee silverskin as wheat flour substitution. J. Food Meas. Charact..

[60] Bresciani L., Calani L., Bruni R., Brighenti F., Del Rio D. (2014). Phenolic composition, caffeine content and antioxidant capacity of coffee silverskin. Food Res. Int..

[61] Hijosa-Valsero M., Garita-Cambronero J., Paniagua-García A.I., Díez-Antolínez R. (2018). Biobutanol production from coffee silverskin. Microb. Cell Factories.

[62] Littardi P., Rinaldi M., Grimaldi M., Cavazza A., Chiavaro E. (2021). Effect of Addition of Green Coffee Parchment on Structural, Qualitative and Chemical Properties of Gluten-Free Bread. Foods.

[63] Iriondo-DeHond A., Rios M.B., Herrera T., Rodriguez-Bertos A., Nuñez F., San Andres M.I., Sanchez-Fortun S., Del Castillo M.D. (2019). Coffee Silverskin Extract: Nutritional Value, Safety and Effect on Key Biological Functions. Nutrients.

[64] Niglio S., Procentese A., Russo M.E., Sannia G., Marzocchella A. (2019). Investigation of Enzymatic Hydrolysis of Coffee Silverskin Aimed at the Production of Butanol and Succinic Acid by Fermentative Processes. BioEnergy Res..

[65] Machado M., Espírito Santo L., Machado S., Lobo J.C., Costa A.S.G., Oliveira M.B.P.P., Ferreira H., Alves R.C. (2023). Bioactive Potential and Chemical Composition of Coffee By-Products: From Pulp to Silverskin. Foods.

[66] Wen L., Álvarez C., Zhang Z., Poojary M.M., Lund M.N., Sun D.-W., Tiwari B.K. (2021). Optimisation and characterisation of protein extraction from coffee silverskin assisted by ultrasound or microwave techniques. Biomass Convers. Biorefin..

[67] Benítez V., Rebollo-Hernanz M., Aguilera Y., Bejerano S., Cañas S., Martín-Cabrejas M.A. (2021). Extruded coffee parchment shows enhanced antioxidant, hypoglycaemic, and hypolipidemic properties by releasing phenolic compounds from the fibre matrix. Food Funct..

[68] Fonseca-García L., Calderón-Jaimes L.S., Rivera M.E. (2014). Capacidad antioxidante y contenido de fenoles totales en café y subproductos del café producido y comercializado en norte de Santander (Colombia). Vitae.

[69] Bondam A.F., Diolinda da Silveira D., Pozzada dos Santos J., Hoffmann J.F. (2022). Phenolic compounds from coffee by-products: Extraction and application in the food and pharmaceutical industries. Trends Food Sci. Technol..

[70] Oliveira L.S., Franca A.S., Mendonça J.C.F., Barros-Júnior M.C. (2006). Proximate composition and fatty acids profile of green and roasted defective coffee beans. LWT–Food Sci. Technol..

[71] Vasconcelos A.L.S., Franca A.S., Glória M.B.A., Mendonça J.C.F. (2007). A comparative study of chemical attributes and levels of amines in defective green and roasted coffee beans. Food Chem..

[72] Castaldo L., Lombardi S., Gaspari A., Rubino M., Izzo L., Narváez A., Ritieni A., Grosso M. (2021). In Vitro Bioaccessibility and Antioxidant Activity of Polyphenolic Compounds from Spent Coffee Grounds-Enriched Cookies. Foods.

[73] Coelho G.O., Batista M.J.A., Ávila A.F., Franca A.S., Oliveira L.S. (2021). Development and characterization of biopolymeric films of galactomannans recovered from spent coffee grounds. J. Food Eng..

[74] Cruz R., Cardoso M.M., Fernandes L., Oliveira M., Mendes E., Baptista P., Morais S., Casal S. (2012). Espresso Coffee Residues: A Valuable Source of Unextracted Compounds. J. Agric. Food Chem..

[75] Vázquez-Sánchez K., Martinez-Saez N., Rebollo-Hernanz M., del Castillo M.D., Gaytán-Martínez M., Campos-Vega R. (2018). In vitro health promoting properties of antioxidant dietary fiber extracted from spent coffee (*Coffea arabica* L.) grounds. Food Chem..

[76] Martinez-Steele E., Khandpur N., Batis C., Bes-Rastrollo M., Bonaccio M., Cediel G., Huybrechts I., Juul F., Levy R.B., Louzada M.L.d.C. (2023). Best practices for applying the Nova food classification system. Nat. Food.

[77] El-Haggar S., Samaha A. (2019). Roadmap for Global Sustainability—Rise of the Green Communities.

[78] Turck D., Bohn T., Castenmiller J., De Henauw S., Hirsch-Ernst K.I., Maciuk A., Mangelsdorf I., McArdle H.J., Naska A., EFSA Panel on Nutrition, Novel Foods and Food Allergens (NDA) (2022). Safety of dried coffee husk (cascara) from *Coffea arabica* L. as a Novel food pursuant to Regulation (EU) 2015/2283. EFSA J..

[79] Duangjai A., Suphrom N., Wungrath J., Ontawong A., Nuengchamnong N., Yosboonruang A. (2016). Comparison of antioxidant, antimicrobial activities and chemical profiles of three coffee (*Coffea arabica* L.) pulp aqueous extracts. Integr. Med. Res..

[80] Iriondo-DeHond A., Martorell P., Genovés S., Ramón D., Stamatakis K., Fresno M., Molina A., Del Castillo M.D. (2016). Coffee Silverskin Extract Protects against Accelerated Aging Caused by Oxidative Agents. Molecules.

[81] Kumar N.S., Hewavitharanage P., Adikaram N.K.B. (1995). Attack on tea by *Xyleborus fornicatus*: Inhibition of the symbiote, *Monacrosporium ambrosium*, by caffeine. Phytochemistry.

[82] Mirón-Mérida V.A., Yáñez-Fernández J., Montañez-Barragán B., Barragán Huerta B.E. (2019). Valorization of coffee parchment waste (*Coffea arabica*) as a source of caffeine and phenolic compounds in antifungal gellan gum films. LWT.

[83] Castro-Díaz R., Silva-Beltrán N.P., Gámez-Meza N., Calderón K. (2025). The Antimicrobial Effects of Coffee and By-Products and Their Potential Applications in Healthcare and Agricultural Sectors: A State-of-Art Review. Microorganisms.

[84] Rebollo-Hernanz M., Cañas S., Taladrid D., Benítez V., Bartolomé B., Aguilera Y., Martín-Cabrejas M.A. (2021). Revalorization of Coffee Husk: Modeling and Optimizing the Green Sustainable Extraction of Phenolic Compounds. Foods.

[85] Král E., Rukov J., Mendes A. (2023). Coffee Cherry on the Top: Disserting Valorization of Coffee Pulp and Husk. Food Eng. Rev..

[86] Martínez J.R.R., Clifford M.N., Sera T., Soccol C.R., Pandey A., Roussos S. (2000). Coffee Pulp Polyphenols: An Overview. Coffee Biotechnology and Quality: Proceedings of the 3rd International Seminar on Biotechnology in the Coffee Agro-Industry, Londrina, Brazil.

[87] Chaves-Ulate C., Rodríguez-Sánchez C., Arias-Echandi M.L., Esquivel P. (2023). Antimicrobial activities of phenolic extracts of coffee mucilage. NFS J..

[88] Farah A., Donangelo C.M. (2006). Phenolic compounds in coffee. Braz. J. Plant Physiol..

[89] Khochapong W., Ketnawa S., Ogawa Y., Punbusayakul N. (2021). Effect of in vitro digestion on bioactive compounds, antioxidant and antimicrobial activities of coffee (*Coffea arabica* L.) pulp aqueous extract. Food Chem..

[90] Esquivel P., Jiménez V.M. (2012). Functional properties of coffee and coffee by-products. Food Res. Int..

[91] Reis R.S., Tienne L.G.P., Souza D.D.H.S., Marques M.D.F.V., Monteiro S.N. (2020). Characterization of coffee parchment and innovative steam explosion treatment to obtain microfibrillated cellulose as potential composite reinforcement. J. Mater. Res. Technol..

[92] Aguilera Y., Rebollo-Hernanz M., Cañas S., Taladrid D., Martín-Cabrejas M.A. (2019). Response surface methodology to optimise the heat-assisted aqueous extraction of phenolic compounds from coffee parchment and their comprehensive analysis. Food Funct..

[93] Maimulyanti A., Nurhidayati I., Mellisani B., Amelia Rachmawati Putri F., Puspita F., Restu Prihadi A. (2023). Development of natural deep eutectic solvent (NADES) based on choline chloride as a green solvent to extract phenolic compound from coffee husk waste. Arab. J. Chem..

[94] Oliveira A., Moreira T.F.M., Paes Silva B., Oliveira G., Teixeira V.M.C., Watanabe L.S., Nixdorf S.L., Leal L.E., Pessoa L.G.A., Seixas F.A.V. (2024). Characterization and bioactivities of coffee husks extract encapsulated with polyvinylpyrrolidone. Food Res. Int..

[95] Yang J., Li Y., Liu B., Wang K., Li H., Peng L. (2024). Carboxymethyl cellulose-based multifunctional film integrated with polyphenol-rich extract and carbon dots from coffee husk waste for active food packaging applications. Food Chem..

[96] Bessada S.M.F., Alves R.C., Costa A.S.G., Nunes M.A., Oliveira M.B.P.P. (2018). *Coffea canephora* silverskin from different geographical origins: A comparative study. Sci. Total Environ..

[97] Munyendo L.M., Njoroge D.M., Owaga E.E., Mugendi B. (2021). Coffee phytochemicals and post-harvest handling—A complex and delicate balance. J. Food Compos. Anal..

[98] Campa C., Doulbeau S., Dussert S., Hamon S., Noirot M. (2005). Qualitative relationship between caffeine and chlorogenic acid contents among wild Coffea species. Food Chem..

[99] Puerta Quintero G. (2011). Composición Química de Una Taza de Café.

[100] Farah A., de Paula Lima J. (2019). Consumption of Chlorogenic Acids through Coffee and Health Implications. Beverages.

[101] Ali A., Zahid H.F., Cottrell J.J., Dunshea F.R. (2022). A Comparative Study for Nutritional and Phytochemical Profiling of *Coffea arabica* (*C. arabica*) from Different Origins and Their Antioxidant Potential and Molecular Docking. Molecules.

[102] Santos É.M., de Macedo L.M., Ataide J.A., Delafiori J., de Oliveira Guarnieri J.P., Rosa P.C.P., Ruiz A.L.T.G., Lancellotti M., Jozala A.F., Catharino R.R. (2024). Antioxidant, antimicrobial and healing properties of an extract from coffee pulp for the development of a phytocosmetic. Sci. Rep..

[103] Zhou H., Gao S.-J., Zhang M.-T., Jia J., Chen F.-X., Chen C.-L., Yang P.-F., Mao J.-L. (2023). Synthesis, configurational analysis and antiviral activities of novel diphenylacrylic acids with caffeic acid as the lead compound. J. Mol. Struct..

[104] Zhang G., Tan Y., Yu T., Wang S., Liu L., Li C. (2021). Synergistic antibacterial effects of reuterin and catechin against *Streptococcus mutans*. LWT.

[105] Majumdar G., Mandal S. (2024). Evaluation of broad-spectrum antibacterial efficacy of quercetin by molecular docking, molecular dynamics simulation and in vitro studies. Chem. Phys. Impact.

[106] Freitas V.V., Borges L.L.R., Vidigal M.C.T.R., dos Santos M.H., Stringheta P.C. (2024). Coffee: A comprehensive overview of origin, market, and the quality process. Trends Food Sci. Technol..

[107] Kar A., Mukherjee S.K., Barik S., Hossain S.T. (2024). Antimicrobial Activity of Trigonelline Hydrochloride Against Pseudomonas aeruginosa and Its Quorum-Sensing Regulated Molecular Mechanisms on Biofilm Formation and Virulence. ACS Infect. Dis..

[108] Woziwodzka A., Krychowiak-Maśnicka M., Gołuński G., Łosiewska A., Borowik A., Wyrzykowski D., Piosik J. (2022). New Life of an Old Drug: Caffeine as a Modulator of Antibacterial Activity of Commonly Used Antibiotics. Pharmaceuticals.

[109] Borrelli R.C., Visconti A., Mennella C., Anese M., Fogliano V. (2002). Chemical Characterization and Antioxidant Properties of Coffee Melanoidins. J. Agric. Food Chem..

[110] Nunes F.M., Coimbra M.A. (2007). Melanoidins from coffee infusions. Fractionation, chemical characterization, and effect of the degree of roast. J. Agric. Food Chem..

[111] Rufián-Henares J.A., de la Cueva S.P. (2009). Antimicrobial Activity of Coffee Melanoidins—A Study of Their Metal-Chelating Properties. J. Agric. Food Chem..

[112] Monente C., Bravo J., Vitas A.I., Arbillaga L., De Peña M.P., Cid C. (2015). Coffee and spent coffee extracts protect against cell mutagens and inhibit growth of food-borne pathogen microorganisms. J. Funct. Foods.

[113] Murthy P.S., Manonmani H.K. (2009). Physico-chemical, antioxidant and antimicrobial properties of Indian monsooned coffee. Eur. Food Res. Technol..

[114] Russell A.D. (1991). Mechanisms of bacterial resistance to non-antibiotics: Food additives and food and pharmaceutical preservatives. J. Appl. Bacteriol..

[115] Prasetya R.C., Fatimatuzzahro N., Ermawati T., Kristina S., Prabaningrum R.R.H. (2024). Antibacterial Activity of Robusta Coffee (*Coffea canephora*) Husk Extract Against *Enterococcus faecalis* and *Phorphyromonas gingivalis*: In Vitro Study. Trends Sci..

[116] Almeida A.A., Farah A., Silva D.A., Nunan E.A., Glória M.B. (2006). Antibacterial activity of coffee extracts and selected coffee chemical compounds against enterobacteria. J. Agric. Food Chem..

[117] Runti G., Pacor S., Colomban S., Gennaro R., Navarini L., Scocchi M. (2015). Arabica coffee extract shows antibacterial activity against *Staphylococcus epidermidis* and *Enterococcus faecalis* and low toxicity towards a human cell line. LWT-Food Sci. Technol..

[118] Castaldo L., Narváez A., Izzo L., Graziani G., Ritieni A. (2020). In Vitro Bioaccessibility and Antioxidant Activity of Coffee Silverskin Polyphenolic Extract and Characterization of Bioactive Compounds Using UHPLC-Q-Orbitrap HRMS. Molecules.

